# Role of RbBP5 and H3K4me3 in the vicinity of Snail transcription start site during epithelial-mesenchymal transition in prostate cancer cell

**DOI:** 10.18632/oncotarget.11549

**Published:** 2016-08-23

**Authors:** Dong Li, Hui Sun, Wen-jing Sun, Hong-bo Bao, Shu-han Si, Jia-lin Fan, Ping Lin, Rong-jun Cui, Yu-jia Pan, Si-min Wen, Xiu-lan Zheng, Xiao-guang Yu

**Affiliations:** ^1^ Department of Biochemistry and Molecular Biology, Harbin Medical University, Harbin, Heilongjiang 150081, P.R. China; ^2^ Department of Ultrasonography, Harbin Medical University Cancer Hospital, Harbin, Heilongjiang 150081, P.R. China; ^3^ Department of Clinical Laboratory, The Second Clinical Medical School of Inner Mongolia University for The Nationalities, (Inner Mongolia Forestry General Hospital), Hulunbuir, Inner Mongolia 022150, P.R. China; ^4^ Department of Neurosurgery, Harbin Medical University Cancer Hospital, Harbin, Heilongjiang 150081, P.R. China; ^5^ Department of Biochemistry and Molecular Biology, Mudanjiang Medical University, Mudanjiang, Heilongjiang 157000, P.R. China

**Keywords:** Snail, H3K4me3, RbBP5, EMT, prostate cancer

## Abstract

EMT (epithelial-mesenchymal transition) occurs in a wide range of tumor types, and has been shown to be crucial for metastasis. Epigenetic modifications of histones contribute to chromatin structure and result in the alterations in gene expression. Tri-methylation of histone H3 lysine 4 (H3K4me3) is associated with the promoters of actively transcribed genes and can serve as a transcriptional on/off switch. RbBP5 is a component of the COMPASS/ -like complex, which catalyzes H3K4me3 formation. In this study, we found that in the process of TGF-Beta1 induced EMT in the prostate cancer cell line DU145, H3K4me3 enrichment and RbBP5 binding increased in the vicinity of Snail (SNAI1) transcription start site. Knocking-down of RbBP5 notably decreased Snail expression and EMT. Recruitment of RbBP5 and formation of H3K4me3 at Snail TSS during EMT depend on binding of SMAD2/3 and CBP at Snail TSS. This study links the SMAD2/3 signal with Snail transcription via a histone modification - H3K4me3. Furthermore, our research also demonstrates that RbBP5 and even WRAD may be a promising therapeutic candidates in treating prostate cancer metastasis, and that DU145 cells maintain their incomplete mesenchymal state in an auto/paracrine manner.

## INTRODUCTION

Prostate cancer (PCa) is the sixth leading cause of cancer-related death in men in the worldwide [1] and the second leading cause in the U.S. [2]. In China, the number of men diagnosed with PCa is increasing dramatically every year [3]. Although most patients survive in the initial stages of prostate cancer after undergoing androgen ablation therapy, if tumor progression, metastasis will lead to poor prognosis and death.

Much evidence has indicated that epithelial- mesenchymal transition (EMT) plays an essential role in cancer metastasis [4, 5]. During EMT, cells lose their epithelial characteristics such as cell polarity and cell-cell contact, and gain mesenchymal features such as motility and a spindle-shaped phenotype [6, 7]. EMT is a reversible and dynamic process, and is evoked by signals from the microenvironment [8–10], for instance, TGF-Beta, Wnt, and TNFα[11, 12]. TGF-Beta is a significant element promoting EMT induction in epithelial cells cancer progression [5]. This factor stimulates cancer cells to become invasive, and leave the primary tumor site, and disseminate to distant sites. Importantly, when a metastatic lesion settled, its cells can re-achieve the epithelial phenotype by mesenchymal- epithelial transition (MET) [6, 13, 14].

Histone modifications act as a significant role in regulating gene expression and chromatin accessibility via altering the chromatin compaction and recruiting other factors such as chromatin remodellers [15–18]. A previous study has demonstrated that a genome-wide H3K9me2/3 re-deposition occurs during a TGF-Beta1(TGFb) induced mouse liver AML12 cell EMT [19]; consistently with this finding, genome-wide histone H3 modifications changes occur during primary prostate cell EMT [20]. In cancer cell EMT, studies have shown that H3 methyltransferase G9a and H3K9me3 are crucial for silencing of E-cadherin (CDH1) during EMT [21, 22].

Interestingly, the expression of epithelial and mesenchymal marker gene, such as E-cadherin and Vimentin, have been reported to be related with histone modifications changes, but the link between expression of EMT associated transcription factors and histone modifications at their gene sites remains unknown [21, 23–25].

Tri-methylation of histone H3 lysine 4 (H3K4me3) is a major chromatin modification in eukaryotes [18, 26]. Modifiers of H3K4me3 involve in various biological processes, including embryonic development [27], stem cell differentiation [28, 29] and cancer [30, 31]. The formation of H3K4me3 is catalyzed by COMPASS/ -like complex. In human, at its core, is composed of either KMT2A/MLL1, KMT2B/MLL2, KMT2C/MLL3, KMT2D/MLL4, SETD1A, or SETD1B associated with WRAD module (WDR5, RBBP5, ASH2L, and DPY30) and other variable partners [32, 33]. Perturbations in H3K4me3-modifying complexes lead to cancer in mammals [34] and lifespan changes in invertebrates [35]. H3K4me3 modification is associated with the promoters of actively transcribed genes and is likely to serve as a transcriptional on/off switch [36]. RbBP5 (Retinoblastoma Binding Protein 5), is one of the conserved “WRAD” components of COMPASS. Ash2L/RbBP5 and MLL1- SET domain make direct contact with the substrates and contribute to the formation of a catalytic center [37].

Snail (SNAI1) belongs to the Snail superfamily of transcription factors, which share a SNAG domain and at least four functional zinc fingers [38]. Snail has been implicated in various processes relating to cell differentiation and survival [38]. Snail plays a activating role in EMT process. Snail performs its function by directly binding to CDH1 promoter and inhibiting transcription via recruiting polycomb repressive complex 2 (PRC2) and G9a; these factors promote methylation of H3K27 and H3K9 which consequently turns off transcription [21, 23]. Snail is up-regulated by several signalling pathway, including the canonical TGF-Beta pathway. Two major downstream factors in the TGF-beta pathway -SMAD2/3, are directly bind the Snail promoter, and promote Snail transcription [39–41], however, whether and how epigenetic mechanisms might contribute to this process remain elusive.

CBP (CREBBP, CREB binding protein) and its paralog, p300 (also called EP300, often referred to collectively as CBP/p300) are transcriptional coactivators of many transcription factors, they catalyze histone acetylation and act as adaptors facilitating DNA binding of general transcription factors [42–44]. Previous research has demonstrated that CBP/P300 act as a co-activator of SMAD2/3 in the regulation of EMT associated TFs transcription in TGF-Beta1 induced cell EMT.

Here, in this study, we show that TGF-Beta1-induced EMT in the prostate cancer cell line DU145, and H3K4me3 enrichment and the binding of COMPASS complex components -WDR5/RbBP5 are increased in the vicinity of Snail transcription start site. Abrogation of RbBP5 suppressed H3K4me3 enrichment and RbBP5 binding at Snail TSS as well as inhibiting EMT process notably. Recruitment of RbBP5 and formation of H3K4me3 at Snail TSS during EMT depend on binding of SMAD2/3 and CBP at Snail TSS. This study connects the SMAD2/3 signal with Snail1 transcription via histone modification - H3K4me3, and demonstrates that RbBP5 sensitizes DU145 to TGF-Beta1. Furthermore, our results indicate that RbBP5 and even WRAD may be promising therapeutic candidates for prostate cancer metastasis treatment. We also show that DU145 cells maintain their incomplete mesenchymal state in an auto/paracrine manner.

## RESULTS

### TGF-Beta1 induced the mesenchymal state in the prostate cancer cell line DU145 by up-regulating Snail

**Figure 1:**
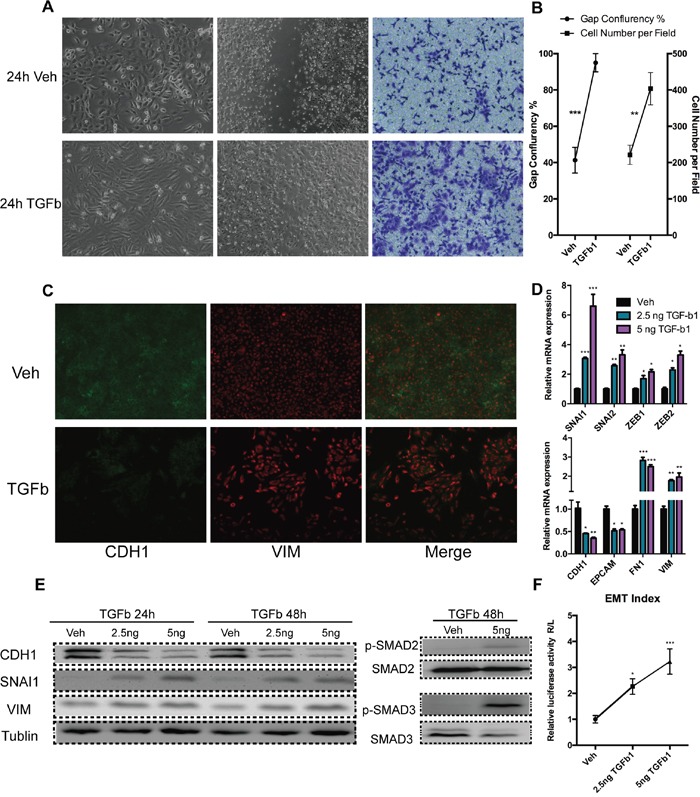
Mesenchymal transition of DU145 cells with incubation of TGF-Beta1 **A.** Bright-phase microscopy (left); wound healing assay (middle); Trans-well migration assay (right): DU145 cells were serum-starved and stimulated with TGF-Beta1 or Veh (PBS). **B.** Results analyses of wound healing (gap confluence) and Transwell migration (cell number per field) assay (40 X). **C.** Immunofluorescence: Expression of adherent junction protein E-cadherin (green) and mesenchymal intermediary filament Vimentin (red, 40 X). **D.** RNA expression of epithelial/ mesenchymal markers and EMT associated transcription factors 24 h post-incubation of TGF-Beta1 in DU145 cells. **E.** Expression of CDH1 (E-cadherin) VIM (Vimentin), and SNAI1 (Snail) and phosphorylation levels of SMAD2 and SMAD3 24/48 h post-incubation TGF-Beta1 at various concentration in DU145 cells. **F.** EMT index: Expression as ratio of Renilla (VIM promoter) and Firefly (CDH1 promoter) luciferase activity, 24 h post-TGF-Beta1 incubation in DU145 cells. (For student's *t-test:* * *p*<0.05, ** *p*<0.01, *** *p*<0.001; # *p*<0.05: one-way *ANOVA)*

We tested the responses of typical prostate cancer cell lines DU145, PC-3, and 22RV1 to TGF-Beta1. Only DU145 exhibited clear morphology changes (Figure 1A and Supplementary Figure S1C, S1D), and its cell migration rate were enhanced (Figure 1A, 1B). Epithelial molecules were down-regulated as well as mesenchymal were expressed (Figure 1C, 1D, 1E), Interestingly, unlike canonical EMT, the key mesenchymal marker Vimentin was constitutively expressed in DU145 cells, and was only slightly up-regulated by TGF-Beta1 (Figure 1C, 1D, 1E). EMT associated transcription factors were expressed under TGF-Beta1 treatment, with Snail (SNAI1) presenting a relatively higher fold-change than the other factors (Figure 1D, 1E). To confirm the predominant role of Snail in DU145 EMT process, we induced EMT employing TGF-Beta1 under rescinding Snail expression by siRNA. These results indicated that abrogation of Snail blocked EMT in DU145 completely (Supplementary Figure S1A, S1B).

Furthermore, as expected, TGF-Beta1 activated SMAD2/3 pathway in DU145 cells (Figure 1E). However, 22RV1, PC-3 cell lines did not exhibit a EMT process after TGF-Beta1 treatment, on the basis of cell morphology (Supplementary Figure S1C, S1D) and their molecular signatures (Supplementary Figure S1E).

To measure the degree of EMT, we designed and applied a dual-luciferase reporter gene system which bearing promoter of CDH1 and VIM. Results indicated that TGF-Beta 1 induced a EMT in DU145 significantly (Figure 1F).

### TGF-Beta1 induced H3K4me3 enrichment in the vicinity of Snail Transcription Start Site (TSS)

**Figure 2:**
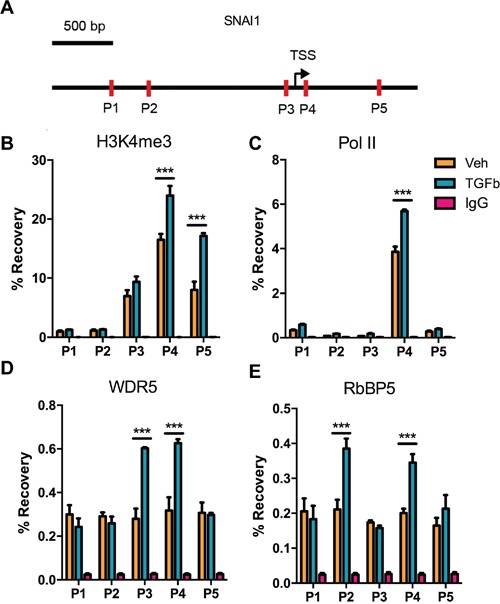
Changes in histone H3 modifications and RNA polymerase II binding at the SNAI1 TSS after TGF-Beta1 induction of EMT in DU145 cells **A.** Schematic depiction of the SNAI1 promoter (−2k − +1k). Primer sets for amplicon for ChIP analyses are indicated by the arrows in the schematic diagram (P1-P5). **B-E.** H3K4me3 enrichment and RNA polymerase II/ WDR5/ RbBP5 binding at corresponding sites (P1-P5), after 24 h of Veh/TGF-Beta1 treatment. TSS: Transcription Start Site.

To assess changes in H3K4me3 enrichment at SNAI1 TSS after TGF-Beta1-induced EMT in DU145 cells, ChIP-qPCR was performed employing primers P1-P5 depicting in the Figure 2A. Results indicated that H3K4me3 modification increased at sites P4 and P5 (Figure 2B). As expected, RNA polymerase II binding at P4 was augmented which is congruent with the increasing of SNAI1 expression during EMT (Figure 2B, 2C). Because COMPASS/COMPASS-like complex catalyze formation of methylated H3K4 in mammal cell, we measured enrichment of two universal components of COMPASS complex- RbBP5 and WDR5. Data demonstrated that WDR5 binding increased at Snail TSS after exposure to TGF-Beta1, as did the binding of RbBP5. (Figure 2D, 2E). Enrichment of WDR5 and RbBP5 proved that H3K4me3 was up-regulated at SNAI1 site from another aspect.

Previous studies have shown that E-cadherin (CDH1) expression is under control of Histone modifications [23, 45], so we next detected the H3K4me3, H3K27me3, and H3K9me3 at CDH1 TSS. Results indicated that only H3K27me3 and H3K9me3 were altered significantly (Supplementary Figure S2), as reported [21, 22], however, there were no clear H3K4me3 modification changes. In addition, TGF-beta1 did not induce bulk level changes in H3K4me3, H3K27me3, or H3K9me3 (Supplementary Figure S2E).

### RbBP5 knockdown restricted EMT progression by blocking Snail expression

**Figure 3:**
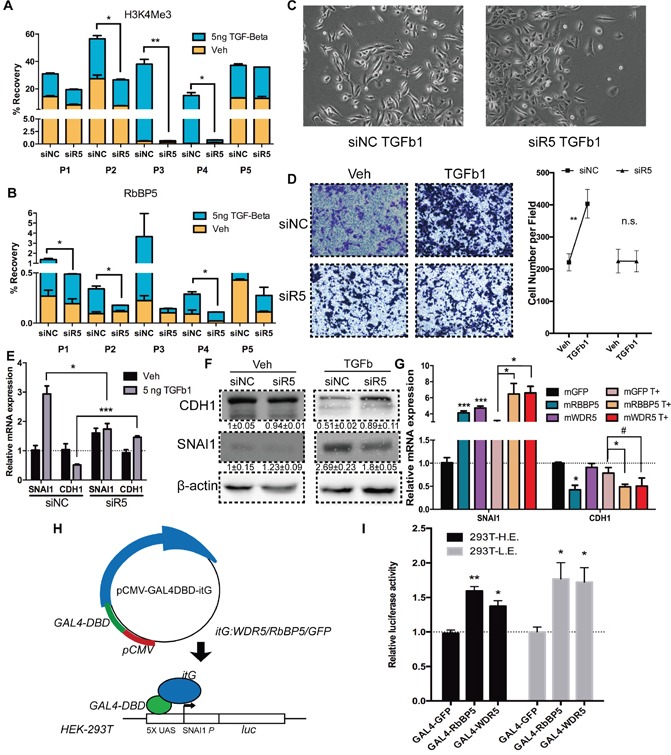
Abrogation of RbBP5 restricted EMT progression via blocking SNAI1 transcription **A.** & **B.** H3K4me3 enrichment and RbBP5 binding at corresponding sites (P1-P5), after 24 h of Veh/TGF-Beta1 treatment, post- transfection of siRbBP5/ siNC. **C.** Bright-phase microscopy: DU145 cells were serum-starved and stimulated with TGF-Beta1 after transfection of siRbBP5/ siNC. **D.** Transwell migration assay and statistical analysis: DU145 cells were serum-starved and stimulated with TGF-Beta1 or Veh (PBS), post-transfection of siRbBP5/ siNC. **E.** & **F.** RNA and Protein expression of SNAI1 and CDH1 in DU145 cells which were serum-starved and stimulated with TGF-Beta1, post-transfection of siRbBP5/ siNC. **G.** RNA expression of SNAI1 and CDH1 in DU145 cells which were serum-starved and stimulated with TGF-Beta1 after transfection of mRbBP5/ mGFP. (T+: TGF-Beta1) **H.** Schematic depiction of the GAL4-UAS reporter system. **I.** Luciferase activity of HE/LE (293T-UAS-SNAI1p-Luc) cell lines transiently transfected with GAL4-GFP/RbBP5/WDR5.

H3K4me3 is distributed around TSS of active transcribed gene, and has be proven to be associated with the initiation of transcription [46]. To confirm the role of H3K4me3 at SNAI1 during TGF-Beta1-induced EMT, we abrogated expression of RbBP5 by RNAi (Supplementary Figure S1F), results substantiate that knocking-down of RbBP5 abolished accumulation of RbBP5 itself and of H3K4me3 at SNAI1 TSS (Figure 3A, 3B, Supplementary Figure S3A). TGF-beta1 induced EMT progression and cell migration were also inhibited (Figure 3C, 3D). Knocking-down of RbBP5 blocked TGF-beta1 induced SNAI1 expression and CDH1 attenuation at mRNA and protein level in DU145 cells (Figure 3E, 3F). Forced expression of RbBP5 or WDR5 mRNA in DU145 resulted in SNAI1 expression increasing in mRNA level, though over-expression of WDR5 was less effective on inhibiting CDH1 expression (p=0.06) (Figure 3G). Bulk-level H3K4me3 modifications changes proved knockdown and over-expression efficiency. (Supplementary Figure S3B).

The alteration of gene expression by H3K4me3, as well as other histone modifications, is dependent on the genomic context [47, 48]. Hence, we engineered a HEK-293T cell line stably integrated with a UAS-SNAI1p-luciferase reporter gene (Figure 3H) [49]. Expression level of luciferase could reflect the various genomic region with different genomic context to a certain degree. Transfection of Gal4-RbBP5/WDR5 were capable of promoting luciferase transcription in representative higher luciferase (HE) and lower expression (LE) clone (Figure 3I, Supplementary Figure S3C). This confirmed that RbBP5/ WDR5 are qualified for promoting Snail transcription hinge on its canonical promoter region (−2000 ~ −1).

### SMAD2/3 recruited RbBP5 to SNAI1 transcription start site via CBP

**Figure 4:**
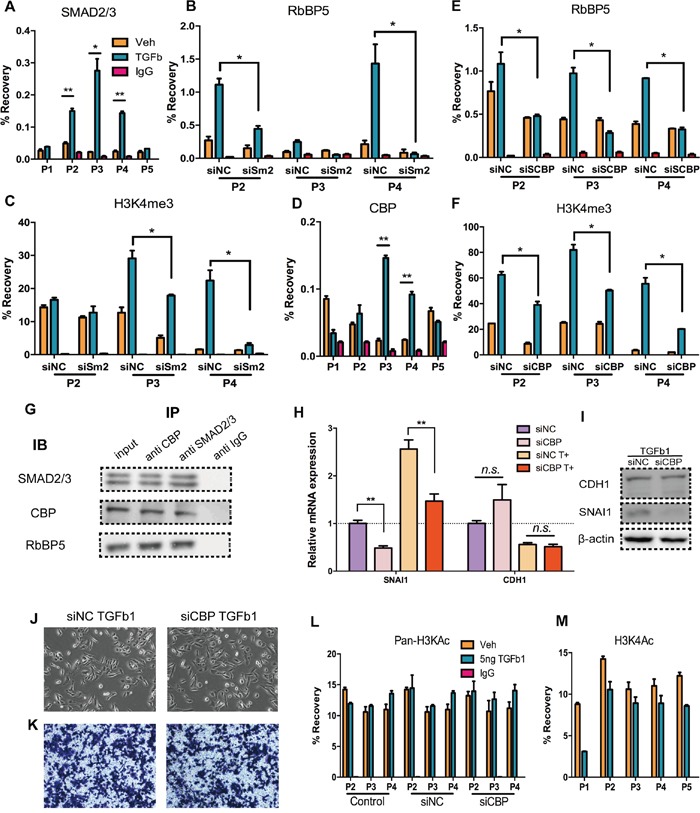
CBP act as bridge mediate the interaction of SMAD2/3 and COMPASS complex **A.** SMAD2/3 binding at corresponding sites (P1-P5), after 24 h of Veh/TGF-Beta1 treatment. **B.** & **C.** RbBP5 binding and H3K4me3 enrichment in corresponding sites (P2-P4), after 24 h of Veh/TGF-Beta1 treatment, post-transfection of siSMAD2/ siNC. **D.** CBP binding in corresponding sites (P1-P5), after 48 h Veh/TGF-Beta1 treatment **E.** & **F.** RbBP5 binding and H3K4me3 enrichment in corresponding sites (P2-P4), after 24 h of Veh/TGF-Beta1 treatment, post-transfection of siCBP/ siNC. **G.** Co-Immunoprecipitation: SMAD2/3, CBP and RbBP5 formed a complex: DU145 cells were incubated with TGF-b1 for 24 h. Antibodies were coupled with protein A/G magnetic beads and detection of IB was performed by a secondary antibody recognizing naive antibodies. Input: 10% lysate. **H, I.** RNA and Protein expression of SNAI1 and CDH1 in DU145 cells which were serum-starved and stimulated with TGF-b1, post-transfected of siCBP/ siNC. **J.** Bright-phase microscopy: Post-transfection of siCBP/ siNC, DU145 cells were serum-starved and stimulated with TGF-b1. **K.** Trans-well migration assay: Post-transfection of siCBP/ siNC, DU145 cells were serum-starved and stimulated with TGF-b1. **L.** Pan- H3Kac (acetyl H3K9, K14, K18, K23 and K27) enrichment at corresponding sites (P2-P4), after 24 h of Veh/TGF-Beta1 treatment, w/o prior transfection of siNC/ siCBP siRNAs. **M.** H3K4ac enrichment at corresponding sites (P2-P4), after 24 h of Veh/TGF-Beta1 treatment.

In EMT, SMAD2/3 directly binds with Snail promoter and promotes its transcription [39, 40] (Figure 4A). To confirm whether H3K4me3 formation at Snail TSS and recruitment of RbBP5 are consequence of SMAD2/3 binding, we performed ChIP assays in SMAD2 knockdown DU145 cells treated with or without TGF-Beta1, interference efficiency was measured by qRT-PCR and western blotting (Supplementary Figure S3D, S3E). Knocking-down of SMAD2 impaired the recruitment of RbBP5 and enrichment of H3K4me3 at Snail TSS (Figure 4B, 4C). Initially, we assumed that SMAD2/3 might directly bind with RbBP5/ WDR5 or any components of COMPASS, but there have been no reports of this phenomenon in the literature. Previous researches has revealed that CBP/p300 function as a bridge facilitating DNA-binding of general transcription factors, and that CBP/p300 bind with both SMAD2/3 and RbBP5/ WDR5 [50, 51]. We measured CBP binding at Snail TSS, and data indicated CBP binding increased after DU145 incubation with TGF-Beta1(Figure 4D).

Next we used siRNA to abrogate CBP expression, and we performed ChIP-qPCR in CBP knockdown DU145 cells treated with or without TGF-Beta1 (Supplementary Figure S3E). Binding of RbBP5 and enrichment of H3K4me3 at Snail TSS post-TGF-Beta1 incubation were down-regulated (Figure 4E, 4F). Co-IP results show that SMAD2/3-CBP-RbBP5 formed a complex (Figure 4G). In addition, after abrogation of CBP, there was no obvious expression alteration of CDH1, whereas a notable decline in Snail expression occurred w/o incubation of TGF-Beta1 (Figure 4H, 4I). Consistently with these observation, TGF-Beta1 treatment on CBP knocking-down cells did not lead apparent changes in morphology or cell migration rate (Figure 4J, 4K). There is a putative explanation: excepting regulating Snail, CBP do directly bind up-steam of CDH1 promoter, maintaining CDH1 expression [52, 53], and CBP also regulate other important genes in EMT [51]. Thus, the over-all effect of CBP-Knockdown could be “no effect” on CDH1 expression.

Furthermore, in addition to its “bridging” function, CBP also catalyzes histone acetylation with intrinsic histone acetyltransferase (HAT) function. To verify whether CBP promote histone acetylation at Snail TSS and whether Snail expression increasing was depend upon histone acetylation, we performed ChIP-qPCR employing an antibody recognizing pan-acetylated histone H3 (acetyl K9 + K14 + K18 + K23 + K27) and an antibody recognizing acetyl-H3K4 (Figure 4L, 4M). Data showed that, no distinct differences of H3K pan-acetylation at Snail TSS after DU145 incubation with TGF-Beta1(Figure 4L), but did show a slight down-regulation of H3K4ac enrichment (Figure 4M), which may result from competition with H3K4me3 [54]. Knocking-down of CBP also did not alter acetylation of H3K at Snail TSS (Figure 4L). These findings confirm that the increasing of Snail expression did not primarily depend on histone H3 lysine acetylation at the Snail TSS, and the underlying mechanism involves CBP's “bridging” function rather than its HAT function.

### Auto/paracrine TGF-beta1 function is important for maintaining the high invasiveness of DU145 cells

**Figure 5:**
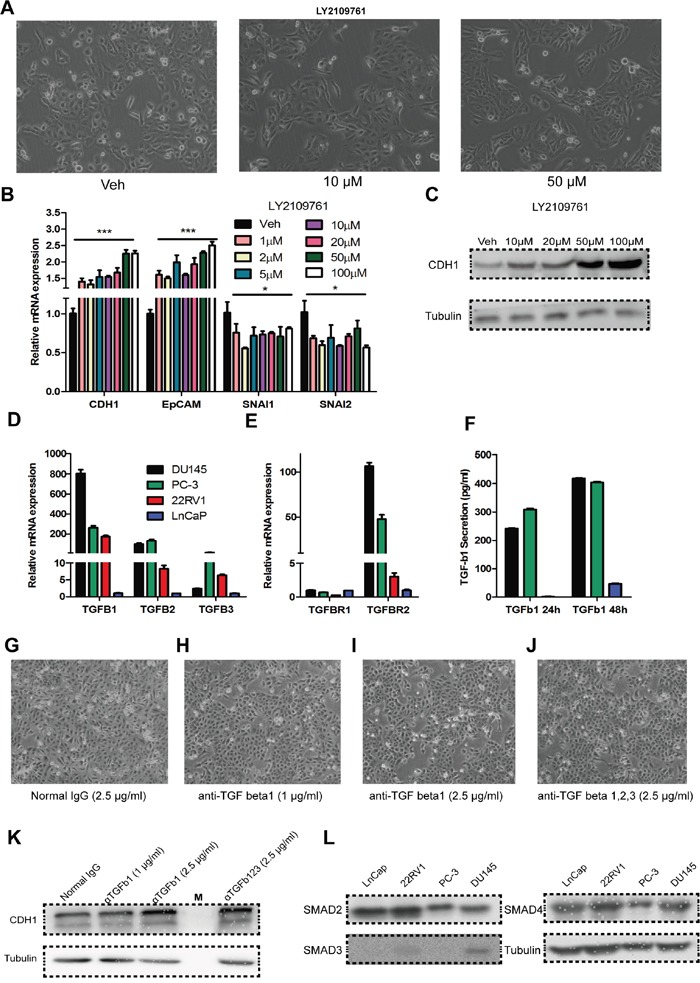
Auto/paracrine function of TGF-beta is important for maintaining the high invasiveness of DU145 cells **A.** Bright-phase microscopy: DU145 cells were incubated with LY2109761(10 μM, 50 μM) or Veh (DMSO). **B.** RNA expression of CDH1, EpCAM, SNAI1 and SNAI2 in DU145 cells incubated with various concentrations of LY2109761 or Veh (DMSO). **C.** CDH1 expression at the protein level in DU145 cells incubated with various concentrations of LY2109761 or Veh (DMSO). **D.** & **E.** RNA expression of TGF Beta1, 2, 3 and TGF Beta receptor 1, 2 in the prostate cancer cell line DU145, PC-3, 22RV1 and Ln-Cap cells. **F.** Secretion of TGF-Beta1 in DU145, PC-3 and Ln-Cap cells for 24/48-hours of culture. **G - I.** Bright-phase microscopy: DU145 cells were incubated with anti-TGF beta1 neutralizing antibody (1 μg/mL, 2.5 μg/mL) or isotype IgG (2.5 μg/mL). **J.** Bright-phase microscopy: DU145 cells were incubated with anti-TGF beta1, 2, 3 neutralizing antibody (2.5 μg/mL). **K.** CDH1 expression at the protein level in DU145 cells incubated with anti-TGF beta1 neutralizing antibody (1 μg/mL, 2.5 μg/mL), anti-TGF beta1, 2, 3 neutralizing antibody (2.5 μg/mL) or isotype IgG (2.5 μg/mL). **L.** Expression of SMAD2, SMAD3 and SMAD4 in LnCap, 22RV1, PC-3 and DU145 cell line, respectively.

To verify whether TGF-Beta1 constitutively maintains the highly invasive status of DU145 cells, we used LY2109761, a TGF-Beta receptor I/II phosphorylation inhibitor, to block TGF-Beta1 pathway activation: epithelia morphology was observed (Figure 5A), with the expected up-regulation of CDH1 (Figure 5B, 5C). We next tested expression of TGF-Beta1/2/3 and TGF-BetaR1/2 in DU145 cells as well as in PC3, 22RV1 and LnCaP cells. Data show that DU145 and PC-3 expressed a much higher level of TGF-Beta1 and TGF-BetaR2 than the other cell lines at mRNA level (Figure 5D, 5E).

Secretion of TGF-Beta1 level was measured in these cell lines by ELISA, and results indicated that a higher level of TGF-Beta1 secretion in DU145 and PC-3 cells. Neutralizing of TGF-Beta1/ TGF-Beta1, 2, 3 in the culture medium caused a similar effect as the inhibition of TGF-BRI/II phosphorylation by LY2109761, in the morphology (Figure 5G–5J) and protein levels (Figure 5K). These indicate that DU145 cells maintain their incomplete mesenchymal state in an auto/paracrine manner

Furthermore, we also measured effects of LY2109761 and neutralizing antibodies on PC-3, 22RV1, and LnCap (data not shown), but there were no apparent alterations in either morphology or molecular signature and these are consistent to unresponsiveness of LnCap and 22RV1 to exotic TGF-Beta1 (Supplementary Figure S1). Lacking of TGF-Beta RI/II, which is congruent with previous studies, may explain the lack of response by LnCap and 22RV1 cells to TGF-Beta1 [8, 55], but the evident disparity in TGF-Beta1 responsiveness between DU145 and PC-3 cells cannot be explained by the receptor expressions. We next detect the expression of SMADs (SMAD2/3/4) in LnCap, PC-3, 22RV1 and DU145 (Figure 5L), results indicated that only DU145 expressed a high level of a down-stem component of canonical TGF-Beta pathway - SMAD3. This may well be a rational explanation for the unresponsiveness of PC-3 to TGF-Beta1. Taken all together, the expression of TGFBR and SMAD3 determine the responsiveness to TGF-Beta1 among DU145, PC3, 22RV1 and LnCaP cells corporately.

## DISCUSSION

Despite significant advances in diagnosing and treating prostate cancer, metastasis continues to be an obstacle of successful therapy and is the main cause of PCa-related death [56]. EMT is a potential mechanism by which tumor cells acquire metastatic features. Implications of EMT encompass enhanced mobility, invasion and resistance to apoptotic stimuli [6, 7, 57]. Furthermore, tumor cells earn cancer stem cell and chemo-resistance properties via EMT [58, 59].

TGF-Beta in the tumor microenvironment is a significant element for EMT induction in epithelial cells cancer progression [5]. This factor stimulates cancer cells to become invasive, leaves the primary tumor site, and disseminate to distant sites. There have also been some reports indicate that the autocrine and paracrine activities of TGF-beta1 are crucial for maintaining mesenchymal and stem cell status in the breast [59, 60]. In our study, we found that TGF-beta1 induces EMT in prostate cancer cell line DU145 by up-regulating SNAI1 (Figure 1), and DU145 maintain themselves in a slight-mesenchymal state by auto/ paracrine of TGF-beta1 activity (Figure 5).

Although the roles of different regulators in mediating EMT and the requirement of chromatin modifiers to coordinate different regulators to mediate EMT have been well demonstrated [21, 22], it is not clear whether or how chromatin modifiers regulate the expression of EMT regulators (EMT-associated TFs, eg., Snail in this study). Whole-genome alterations of histone modifications and DNA modifications during EMT procession have previously been reported, unfortunately, the EMT-associated TFs have not been identified [19, 20]. The COMPASS-/like complex (also known as Complex of Proteins Associated with Set1) has been demonstrated to be a histone H3 lysine 4 (H3K4) methyltransferase [34]. RbBP5 is one of the conserved “WRAD” components of COMPASS, and in humans, the RbBP5-ASH2L heterodimer is the minimal structural unit that interacts with and activates all MLL family histone methyltransferases [37, 61]. These findings may suggest that RbBP5 is more important than other components in COMPASS.

In DU145 cells, we observed TGF-Beta1 induced a H3K4me3 enrichment raising in the vicinity of Snail Transcription Start Site, accompanied by the increasing of Snail transcription (Figure 2). But TGF-beta1 did not induce bulk level changes in H3K4me3 (Supplementary Figure S2). Recruitment of RbBP5 and formation of H3K4me3 at Snail TSS which are mediated by SMAD2/3 were required for the increase of Snail expression and successful EMT progression during TGF-beta1 induced DU145 EMT (Figure 3, 4).

CBP (CREBBP, CREB binding protein) and its paralog p300 (also called EP300) are transcriptional coactivators for many important transcription factors. They function by acting as bridges facilitating DNA binding of general transcription factors and by relaxing chromatin through their intrinsic histone acetyltransferase (HAT) activity [42–44]. Under specific circumstances, CBP and p300 play distinct roles; however, their functions largely overlap [62]. In TGF-Beta1-induced EMT, CBP has been found to act as a co-activator of SMAD2/3 in promoting the transcription of EMT-associated TFs (e.g., Snail) [51], and some evidence has suggested that CBP/p300 also bind with MLL/WDR5/RbBP5, serving as a scaffold to recruit them to transcription start sites and promote gene transcription [50, 63, 64]. From our data, although CBP binding at the SNAI1 TSS increased following TGF-Beta1 treatment (Figure 4D), there were no significant enrichment changes in pan H3Kac (acetyl H3K9, K14, K18, K23 and K27) at the SNAI1 TSS (Figure 4L), and knocking-down of CBP did not change pan H3Kac modifications at Snail TSS (Figure 4L). But there was slight down-regulation of H3K4ac after cell exposure to TGF-beta1 (Figure 4M), probably owing to competition between H3K4me and H3K4ac [54]. However, CBP knockdown abrogated the recruitment of RbBP5 and the enrichment of H3K4me3 at the Snail TSS, as well as Snail expression. These findings demonstrate that at the Snail TSS, CBP serves as a “bridge” connecting SMAD2/3 with COMPASS, but not as a HAT.

**Figure 6:**
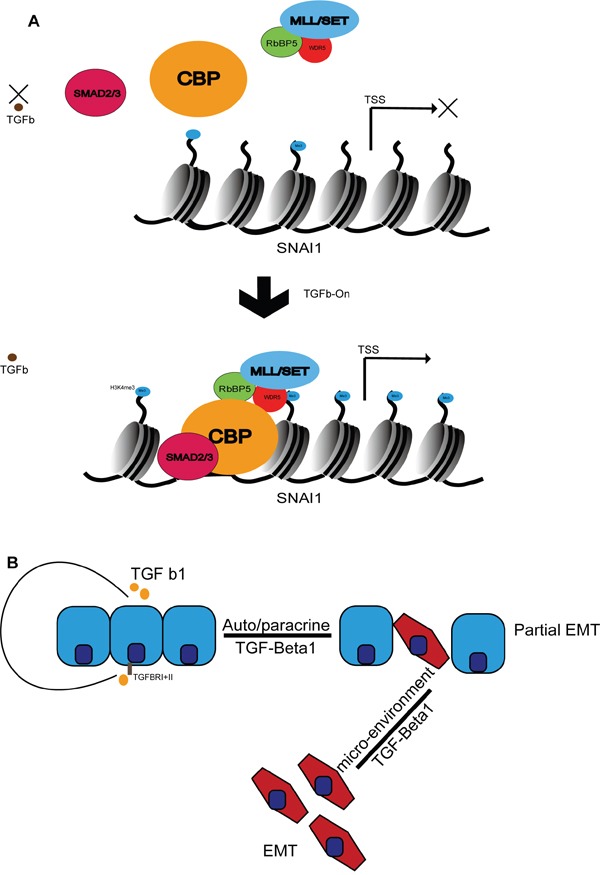
Schematic depiction of putative model of DU145 EMT **A.** A proposed model highlighting TGF-Beta1-SMAD2/3-CBP-COMPASS signal transduction from the TGF-Beta receptor onto chromatin, triggering Snail expression. **B.** Schematic depiction of TGF-beta1 auto/paracrine model of DU145.

Taken all together, our findings indicate that TGF-Beta1 induces DU145 into mesenchymal state and DU145 maintain themselves in a slight-mesenchymal state by auto/ paracrine actions of TGF-beta1. H3K4me3 is deposited at Snail TSS after TGF-Beta1 incubation of DU145, and TGF-Beta1-SMAD2/3-CBP-COMPASS transduce this signal from TGF-Beta receptor onto chromatin (Figure 6). Our study indicates that RbBP5 and even WRAD may be promising therapeutic candidates for prostate cancer metastasis treatment.

## MATERIALS AND METHODS

### Materials

Antibodies information can be found in Supplementary Table S1. Lipofectamine® 3000, RNAiMAX, MessengerMAX, Opti-MEM® reduced serum media, and SYBR® Select Master Mix, were obtained from Invitrogen® (Thermo Fisher, Life-Technologies, Carlsbad, CA, USA). RPMI-1640, DMEM medium, and FBS were obtained from Gibco® (Thermo Fisher, Life-Technologies, Carlsbad, CA, USA). PrimeScript® RT reagent Kit with gDNA Eraser was obtained from Takara bio (Dalian, China). TGF-Beta1 was purchased from PeproTech® (London, UK). mMESSAGE mMACHINE® T7 ULTRA Transcription Kit was obtained from Ambion® (Thermo Fisher, Life-Technologies, Carlsbad, CA, USA) LY2109761(Cat.#S2704) was obtained from Selleck (Houston, TX, USA)

SimpleChIP® Plus Enzymatic Chromatin IP Kit-Magnetic Beads (#9005) were purchased from Cell Signaling Technology (Danvers, MA, USA), and Pierce® Crosslink Magnetic IP/Co-IP Kit(#88805) came from Thermo Fisher Scientific (Waltham, MA, USA). siRNAs for RbBP5, SNAI1, CBP, SMAD2 were synthesized by Ribo Bio(Guangzhou, China). For their sequences, see the Supplementary Table S2.

### Plasmids construction and *in vitro* transcriptions

The ORF of WDR5 and RbBP5 were amplified from cDNA of DU145 cells and cloned into pCMV (Clontech, Mountain View, CA, USA), and fused with Flag, and HA tag. The pGL 4.35-SNAI1 (9x UAS-SNAI1-Luc) was constructed base on pGL 4.35 (Promega, Madison, WI, USA): promoter of SNAI1 (-2000~-1) was amplified from genomic DNA of DU145 cell and cloned into pGL 4.35 with Hind III digestion. GAL4-RBBP5/WDR5 were constructed on the basis of pCMV (Clontech): GAL4-DBD(GAL4) was amplified from pGBKT7 (Clontech), and an IgA linker sequence was added to the 3′ site by primers; WDR5 and RBBP5 were amplified from cDNA of DU145 cells. Promoter of CDH1(-2000~-1) and VIM(-2000~-1) were amplified from genomic DNA of DU145 cell and cloned into pGL3-basic(luc) and pRL-null(Rluc) to form pGL3-CDH1 and pRL-VIM. All constructs were employed with In-Fusion® Clone Kit (Clontech).

Capped and tailed mRNA were generated by mMESSAGE mMACHINE® T7 ULTRA Transcription Kit (Ambion®), as per manufacturer's recommendations. Primer sequence are describing in Supplementary Table S3.

### Cell culture and transfection

Human Prostate cancer cell line DU145 cells and HEK-293T cells were obtained from American Type Culture Collection (ATCC, Manassas, VA, USA). PC-3, Ln-Cap, and 22RV1 were obtained from Cell Resource Center, IBMS, CAMS/PUMC (China). DU145, Ln-Cap, and 22RV1 were maintained in RPMI-1640 medium, as well as HEK-293T, PC-3 were maintained in DMEM medium. All culture media were supplemented with 10% fetal bovine serum (FBS) (Gibco, Thermo Fisher Scientific) and 100U/ml penicillin/streptomycin (Gibco, Thermo Fisher Scientific), at 37 °C in humidified atmosphere (5% CO2/95% air). Cells were incubated in 2.5 or 5 ng/ml TGF-Beta1 or Veh (0.1% BSA in PBS) with 0.5% FBS medium, after 12h of serum starvation.

DNA transfection was performed by using Lipofectamine® 3000 at 80% cell confluence. For siRNA transfection (100 nM), Lipofectamine® RNAiMAX was utilized, at 50% cell confluence. Transfections were performed according to manufacturer's recommendations in 6-well plate. mRNA transfection was employed Lipofectamine® MessengerMAX. At 24 h post-transfection, Cells were trypsinized and seeded to two 6-well plates, and then subjected to TGF-Beta1 treatment.

The pGL 4.35-SNAI1 (9x UAS-SNAI1-Luc) plasmid was stably integrated into the HEK-293T cells and selected by Hygromycin B (200 μg/ml) for 14 days. Two representative clones (one for high expression and one for low) were selected from the 15 picked single clones.

### RNA extraction and real-time PCR

For real-time PCR analyses, total RNA was extracted from cells using Trizol® reagent (Ambion®, Life-Technologies) and this was followed by column based RNA purification method (TIANGEN, Beijing, China) as per the manufacturer's instructions. Clearance of DNA contamination in RNA and cDNA synthesis was performed using the PrimeScript® RT reagent Kit with gDNA Eraser according to the manufacturer's instructions (TaKaRa Bio). Real-time PCR was subsequently performed using the ABI-7500 System employing SYBR® Select Master Mix (Applied Biosystems®, Life-Technologies). Primer sequences are describing in Supplementary Table S4.

### Chromatin immunoprecipitation (ChIP)

ChIP assays were performed according to manufacturer's recommendations of Cell Signaling Technology (#9005). Briefly, 24 h after incubation with TGF-Beta1/Veh, 4,000,000 cells were collected and cross-linked in 1% formaldehyde. Cell nuclei were prepared and chromatin was digested with micrococcal nuclease, then sonicated to break nuclear membrane. Following an overnight incubation with antibodies, 30 μl of Protein G Magnetic Beads was added at 4°C for 2 h. Bound DNA–protein complexes were eluted and crosslinks were reversed after a series of washes. Real-time PCR was subsequently performed using the ABI-7500 System employing SYBR® Select Master Mix (Applied Biosystems, Life-Technologies). Primer sequences are describing in the Supplementary Table S4.

### Co-immunoprecipitation (Co-IP)

Co-IP assays were performed according to manufacturer's recommendations of Thermo Fisher Scientific (#88805). In brief: Antibodies (5 μg/ reaction) were first coupled with Protein A/G Magnetic Beads in a Lysis/Wash Buffer, and cross-linked using DSS (disuccinimidyl suberate). Magnetic Beads then washed and incubated cell lysate 2 h at room temperature. After incubation with lysate from 4,000,000 cells, beads were again washed and protein eluted using a low PH elution buffer. A negative control antibody (isotope IgG) was to assess nonspecific binding received the same treatment as the Co-IP samples, IP samples were analyzed by Western blotting.

### Immunofluorescence

Cells were rinsed three times with PBS and fixed with 4% paraformaldehyde, and then permeabilized with 0.1% Triton X-100. After being blocked in 1% BSA for 2 hours, cells were incubated with primary antibody at 4°C overnight, washed and then incubated for 2 hours with the secondary antibody at room temperature. Washed cells (3 times; PBS containing 0.02% Tween 20) were observed at a fluorescence microscope (Olympus IX71, Japan). Acquisition parameters, shutters, filter positions and focus were controlled by the IX71 software.

### Western-blotting assay

Whole-cell lysates preparation and western blot analysis were carried out as described previously. In brief: cells were harvested and washed in ice-cold PBS, then the protein concentration of the extracts was determined using bicinchoninic acid reagent (BCA) (Thermo Fisher Scientific).

Equal quantities of protein (30 μg per lane for EMT associated immunogen, 10 μg per lane for histone detection) were loaded, separated using 12% SDS-PAGE(15% for Histone separation) and transferred onto nitrocellulose membranes. Subsequent to being blocked with 5% non-fat milk, membranes were incubated with the primary antibodies at 4 °C, overnight. Subsequent to incubation with the corresponding secondary antibody, immune complexes were detected using ECL plus western blotting reagents (Thermo Fisher Scientific). The expression levels of β-actin/ Tubulin or total histone H3 was monitored as the internal control, and band intensities were normalized to that of β-actin/Tubulin or total histone H3.

### Wound healing assay

DU145 cells were seeded in 6-well plates to achieve 90% confluence. Twenty-four hours after TGF-b1 treatment, a vertical wound was created using a 200-μl pipette tip. Then, the cells were washed with PBS for three times and medium without serum was added into the wells. After 24-h incubation, the wound was observed and random fields in each well were selected for imaging. The images were analyzed by ImageJ and the distance of wound closure was used to estimate the migration ability.

### Migration assay

Cell migration and invasive ability were evaluated using 24-well Tran swell plates(Corning). For migration assessment, 24 h after TGF-b1 treatment, 200,000 cells per well were seeded into the top chamber and maintained in serum-free medium. Medium (600 μl) containing 10% FBS was added into the bottom chamber. After being incubated for 6 h at 37 °C, cells that migrated through the pore polycarbonate membrane were fixed with methanol and stained with 0.05% crystal violet. Then, the migrated cells were observed and images were captured using microscopy.

### Luciferase assay

For EMT index: pGL3-CDH1(500 ng) and pRL-VIM (500 ng) were co-transfected into DU145 cell at 200,000 cells per well in a 12-well plate. Forty-eight hours post transfection, cells then were subjected to the TGF-Beta1 treatment. After 24 h of incubation of TGF-Beta1/Veh Firefly and Renilla luciferase activities were measured consecutively with the Dual-Luciferase reporter assay system using a luminometer (both from Promega). The EMT index is expressed as ratio of firefly luciferase(RLU) and Renilla luciferase (RLU).

For pGL 4.35-SNAI1: Two representative 293T- pGL 4.35-SNAI1 clones (one for high expression and one for lower) were seeded into 12-well plate at 200,000 cells per well. GAL4-RBBP5 or GAL4-WDR5 (1ug) was transfected into both clones. After 24 h incubation, luciferase reporter activities were measured in whole cell lysates using the Luciferase Assay System and a luminometer (both from Promega). All experiments were performed in biological and technical triplicates and normalized for protein concentration (BCA).

### Statistical analysis

Each experiment was repeated in triplicate. Statistical analyses (student's *t-test/ ANOVA*) were performed by using Microsoft EXCEL (2016 for OSX 10.11), p<0.05 was considered significant, and results are expressed as mean ± SEM. (* p<0.05, ** p<0.01, *** p<0.001).

## SUPPLEMENTARY FIGURES AND TABLES


