# Supplementary material for: Role of RbBP5 and H3K4me3 in the vicinity of Snail transcription start site during epithelial-mesenchymal transition in prostate cancer cell
Source: Oncotarget. 2016 Aug 23;7(40):65553–67. doi: 10.18632/oncotarget.11549 (PMC5323174; doi:10.18632/oncotarget.11549)
Supplement: Supplementary file 1 [file oncotarget-07-65553-s001.pdf]

Role of RbBP5 and H3K4me3 in the vicinity of snail transcription start site during epithelial- mesenchymal- transition in prostate cancer cell

SUPPLEMENTARY FIGURES AND TABLES

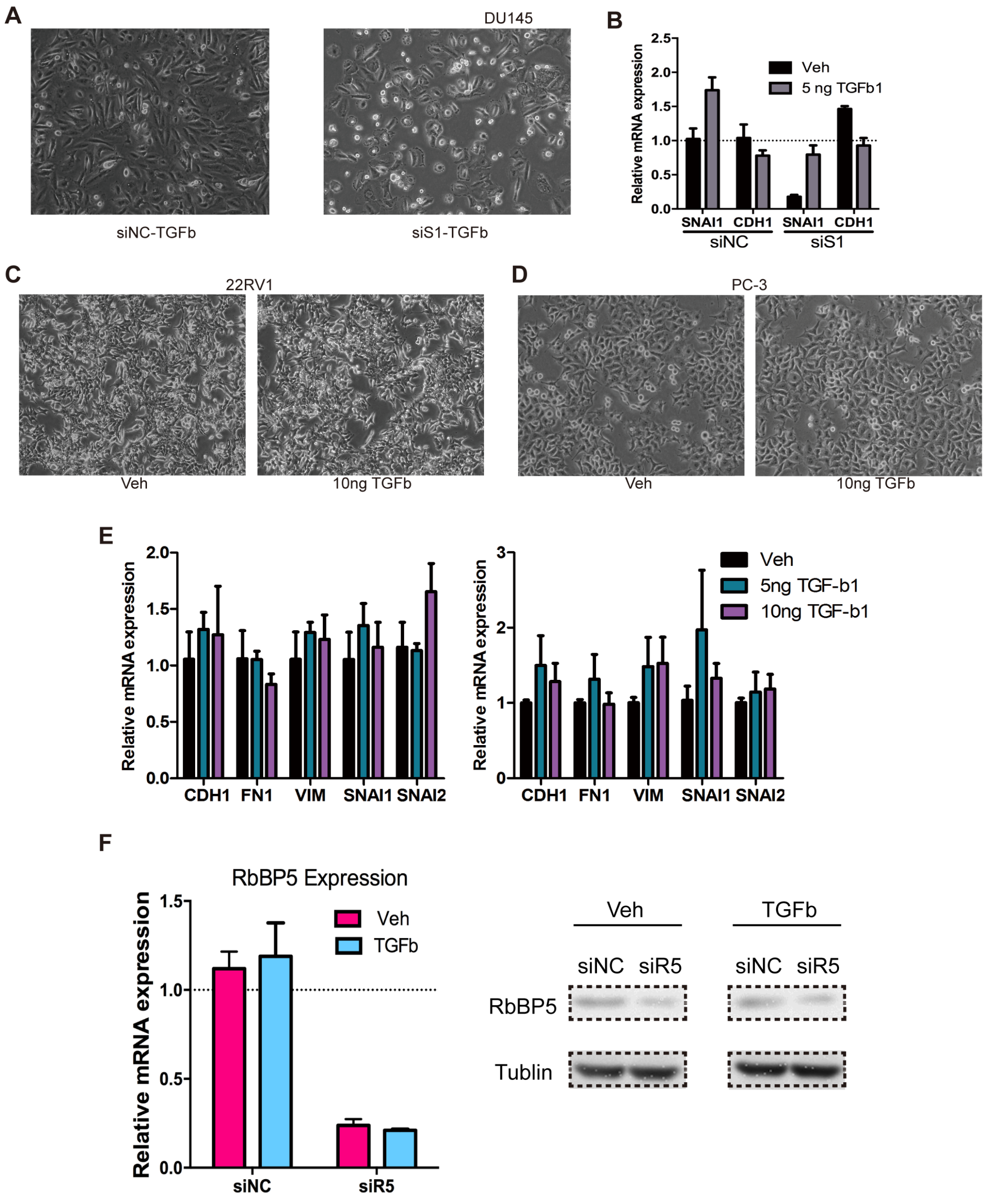

Supplementary Figure S1: A. Bright-phase microscopy: DU145 cells were serum-starved and stimulated with TGF-Beta1, after transfection of siSNAI1/siNC. **B.** SNAI1 and CDH1 mRNA expression of DU145 cells which were serum-starved and stimulated with TGF-Beta1 after transfection of siSNAI1/ siNC. **C & D.** Bright-phase microscopy: 22RV1 and PC-3 cells were serum-starved and stimulated with TGF-Beta1 or Veh (PBS) **E.** Expression of EMT molecular signatures: 22RV1 and PC-3 cells were serum-starved and stimulated with TGF-Beta1 or Veh (PBS) **F.** Knocking-down efficiency of RbBP5 at RNA and protein level w/o TGF-Beta1 incubation.

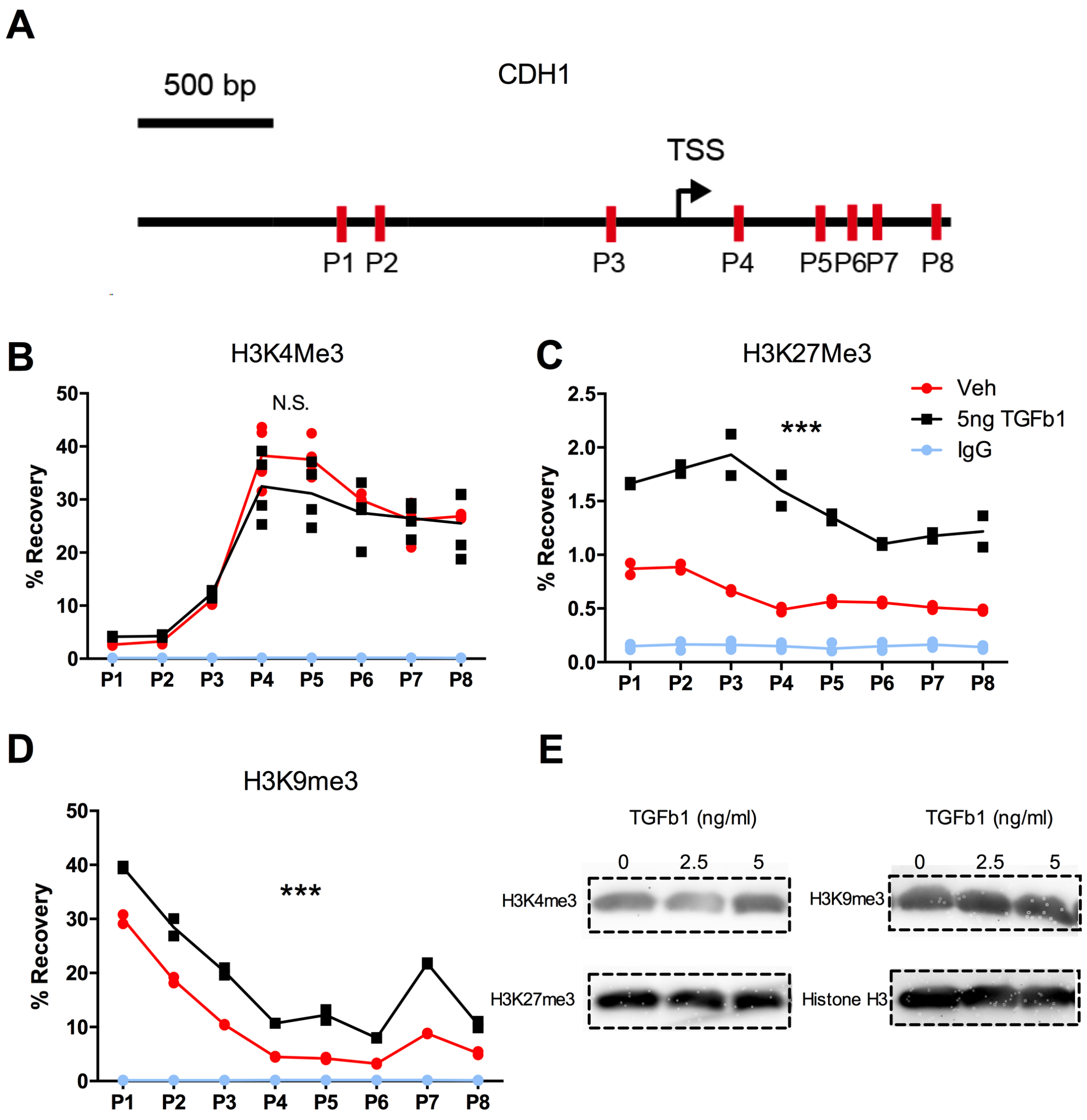

Supplementary Figure S2: Changes in histone H3 modifications at the CDH1 TSS after TGF-Beta1 induction of EMT in DU145 cells. A. Schematic depiction of the CDH1 promoter (-2k - +1k). Primer sets for ChIP amplicon analysis are indicated by the arrows in the schematic diagram (P1-P8). **B-D.** H3K4me3, H3K27me3 and H3K9me3 enrichment at corresponding sites (P1-P8) after 24 h of Veh/TGF-Beta1 treatment. **E.** Bulk level changes in H3K4me3, H3K27me3, H3K9me3 and total H3 in DU145 cells after 24 h of Veh/TGF-Beta1 treatment. (*** p<0.001; 0.001 for P1-P8 amplicons.)

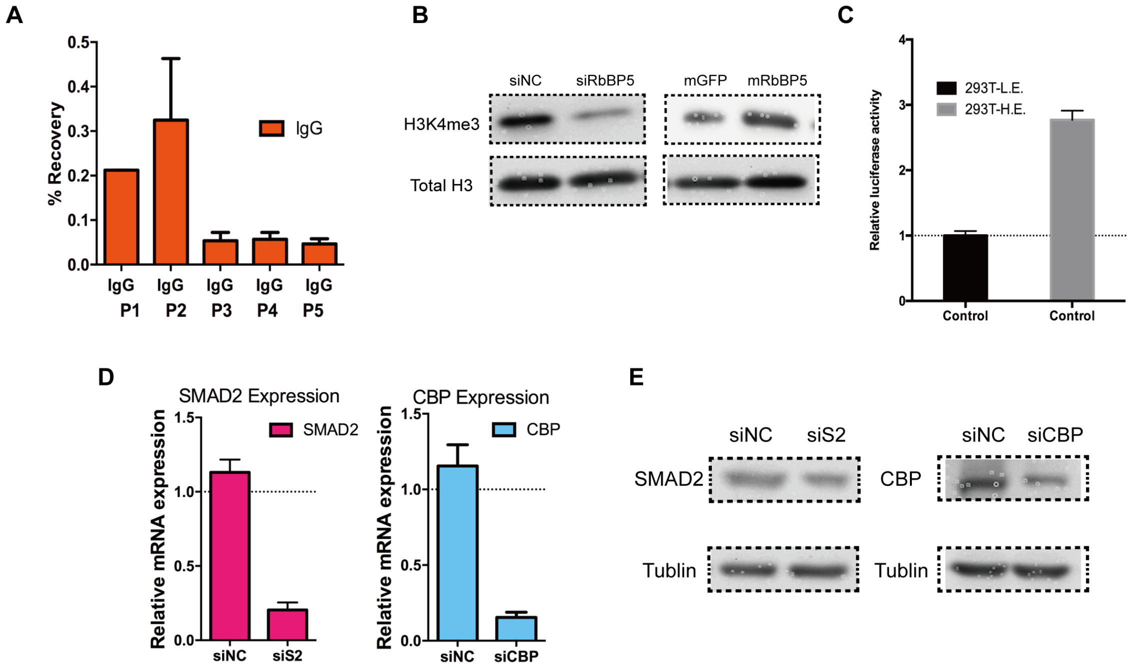

Supplementary Figure S3: A. Amplification results of isotype IgG in DU145 cells (ChIP). **B.** Bulk level modifications of H3K4me3, and total H3 in DU145 cells transiently transfected with siRbBP5/ siNC or mRbBP5/mGFP. **C.** Relative luciferase activity of HE/LE (293T-UAS-SNAI1p-Luc) cell lines **D.** Knocking-down efficiency of SMAD2 and CBP at RNA level. **E.** Knocking-down efficiency of SMAD2 and CBP at protein level.

Supplementary Table S1: Antibodies

Antibody	Catalog #	Supplier	Application	Dilution
E-Cadherin	#3195, rabbit mAb	Cell Signaling Technology	Western blot	1:1000
E-Cadherin	ab76055, mouse mAb	Abcam	Immunofluorescence	1:100
Vimentin	#5741, rabbit mAb	Cell Signaling Technology	Western blot/ Immunofluorescence	1:1000/ 1:100
Snail	#3879, rabbit mAb	Cell Signaling Technology	Western blot	1:1000
Smad2/3	#8685, rabbit mAb	Cell Signaling Technology	Western blot/ Co-IP/ ChIP	1:1000/ 1:100/1:100
p-SMAD2 (Ser465/467)	#3108, rabbit mAb	Cell Signaling Technology	Western blot	1:1000
p-SMAD3 (Ser423/425)	#9520, rabbit mAb	Cell Signaling Technology	Western blot	1:1000
SMAD2	#5339, rabbit mAb	Cell Signaling Technology	Western blot	1:1000
SMAD3	#9523, rabbit mAb	Cell Signaling Technology	Western blot	1:1000
WDR5	#13105, rabbit mAb	Cell Signaling Technology	Western blot/ ChIP	1:1000/ 1:50
RbBP5	#13171, rabbit mAb	Cell Signaling Technology	Western blot/ ChIP	1:1000/ 1:50
RNA Pol II CTD	#2926, rabbit mAb	Cell Signaling Technology	Western blot/ ChIP	1:1000/ 1:50
H3K4me3	ab8580, rabbit pAb	Abcam	Western blot/ChIP	1:1000/ 1:100
H3K9me3	ab8898, rabbit pAb	Abcam	Western blot/ChIP	1:1000/ 1:100
H3K27me3	ab8898, rabbit pAb	Abcam	Western blot/ChIP	1:1000/ 1:100
Pan- H3Kac(acetyl K9 + K14 + K18 + K23 + K27)	ab47915, rabbit pAb	Abcam	ChIP	1:100
CBP	ab2832, rabbit pAb	Abcam	Western blot/ Co-IP/ ChIP	1:1000/ 1:100/1:100
TGF-Beta 1, 2, 3	MAB1835, mouse mAb	R&D	Neutralization	up tp 2.5ug/ mL
TGF-Beta 1	MAB240, mouse mAb	R&D	Neutralization	up to 2.5ug/ mL
Beta- actin	sc-47778, mouse mAb	Santa Cruz	Western blot	1: 500
Alpha- tublin	66031-1-Ig, mouse mAb	Protein tech.	Western blot	1: 500
Alexa Fluor® 488 donkey anti–mouse	A-21202	Life Technologies	Immunofluorescence	1:500
Alexa Fluor® 555 donkey anti–rabbit	A-31572	Life Technologies	Immunofluorescence	1:500
Goat Anti-Rabbit IgG (H+L) HRP	A16104	Life Technologies	Western blot	1:2000
Goat Anti-Mouse IgG (H+L) HRP	A16078	Life Technologies	Western blot	1:2000
Clean-Blot ™ IP Detection Reagent (HRP)	21230	Thermo Fisher	Western blot	1:500

Supplementary Table S2: Sequences of siRNAs

Gene	Sequnence(5'-3')
siWDR5	CGGUGUGUCUACAGCACUA
siRBBP5	GGUGUCGGUAAUUCAGUUA
siSNAI1	GGAAGCAGGAAGAGGAGAU
siCBP	UGGAACAAGGUUCCCACUG
siSMAD2	AACAGGCCUUUACAGCUUCUC
siNC	Due to the policy of Ribo Bio, the sequence cannot be display here.

Supplementary Table S3: Primers for Constructions

Construction description	Sequnence(5'-3')
pCMV-Flag-WDR5 F	GATCTCGAGCTCAAGCATGGATTACAAGGATGACGACGA TAAGGCGACGGAGGAGAAG
pCMV-Flag-WDR5 R	TGATTATGATCTAGA TTAGCAGTCACTCTTCCACAG
pCMV-HA-RbBP5 F	GATCTCGAGCTCAAGCATGTACCCATACGATGTTCCAGATT ACGCTAACCTCGAGTTGCTGGA
pCMV-HA-RbBP5 R	TGATTATGATCTAGATCATAACAGTTCTGAGATTGC
pGL4.35-SNAI1 F	tctgcgatctaagtaagctTCTCACCTCCTCATCAACTCT
pGL4.35-SNAI1 R	cagtaccggattgccaagctTGCAGCAGCGCCGCC
HA-GAL4-DBD-Linker F	GATCTCGAGCTCAAGCATGTACCCATACGATGTTCCAG ATTACGCTAAGCTACTGTCTTCTATCGAACAAG
GAL4-DBD-Linker R	tggagagggagtaggtggtgtcgacggaatcgcCGATACAGTCAACTGTCTTTGACCT
IVT-T7p-WDR5 F	GCTAATACGACTCACTATAGGGCGCCACCATGGATTACAA GGATGACGACGATAAGGCGACGGAGGAGAAG
IVT-T7p-WDR5 R	TGATTATGATCTAGATTAGCAGTCACTCTTCCACAG
IVT-T7p-RbBP5 F	GCTAATACGACTCACTATAGGGCGCCACCATGTACC CATACGATGTTCCAGATTACGCTAACCTCGAGTTGCTGGA
IVT-T7p-RbBP5 R	TGATTATGATCTAGATCATAACAGTTCTGAGATTGC

Supplementary Table S4: Primers for detection

Gene	Sequnence(5'-3')
E-Cadherin F	TACACTGCCCAGGAGCCAGA
E-Cadherin R	TGGCACCAGTGTCCGGATTA
Vimentin F	TGAGTACCGGAGACAGGTGCAG
Vimentin R	TAGCAGCTTCAACGGCAAAGTTC
Snail F	AGTTTACCTTCCAGCAGCCCTAC
Snail R	GACAGAGTCCCAGATGAGCATT
Fibronectin 1 F	CAGTGGGAGACCTCGAGAAG
Fibronectin 1 R	TCCCTCGGAACATCAGAAAC
WDR5 F	CCGTTTGTGTCTTGGGAGTT
WDR5 R	CACTTGCCACAACCATTCAC
RBBP5 F	CATCTTTTGATAGGCGAGGGG
RBBP5 R	GTTCCAGTTGTCACTCTGAAGG
EpCAM F	CTGGCCGTAAACTGCTTTGT
EpCAM R	AGCCATTCATTTCTGCCTTC
GAPDH F	ACCCAGAAGACTGTGGATGG
GAPDH R	CAGTGAGCTTCCCGTTCAG
TGFBR1 F	GCTGTATTGCAGACTTAGGACTG
TGFBR1 R	TTTTTGTTCCCACTCTGTGGTT
TGFBR2 F	GTAGCTCTGATGAGTGCAATGAC
TGFBR2 R	CAGATATGGCAACTCCCAGTG
TGFB1 F	CAATTCCTGGCGATACCTCAG
TGFB1 R	GCACAACTCCGGTGACATCAA
TGFB2 F	CAGCACACTCGATATGGACCA
TGFB2 R	CCTCGGGCTCAGGATAGTCT
TGFB3 F	ACTTGCACCACCTTGGACTTC
TGFB3 R	GGTCATCACCGTTGGCTCA
ChIP Primers	
SNAI1 P1 F	TAAATTGACACGGGACGGGG
SNAI1 P1 R	CTGGTTCTAGCTGGAGAGCG
SNAI1 P2 F	GAACGGTGAGACCTGTGAGG
SNAI1 P2 R	GTAACACGGCTCCATAGGGG
SNAI1 P3 F	TACTTAAGGGAGTTGGCGGC
SNAI1 P3 R	CGCAGAAGAACCACTCGCTA
SNAI1 P4 F	GAACGGTGAGACCTGTGAGG
SNAI1 P4 R	GTAACACGGCTCCATAGGGG
SNAI1 P5 F	GGAACCTGGTCTGTCCTGTG
SNAI1 P5 R	CAACAAACATGAGCCCACCG
CDH1 P1 F	CCACAACAGCATAGGGAGACA
CDH1 P1 R	CTAGGTCAGGACCACCTCCC
CDH1 P2 F	TTCCCATTAGGAGGGTGGAGA
CDH1 P2 R	TTCATGGGTTAGTGAGTCAGC
CDH1 P3 F	CAGTGGAATCAGAACCGTGC
CDH1 P3 R	CTCGCATAGACGCGGTGA
CDH1 P4 F	GGATCCCCTGACTTGCGAGG
CDH1 P4 R	GCTCCTCAGGACCCGAACTT
CDH1 P5 F	GCCATGCCAAGAAAGGTCGT
CDH1 P5 R	CCACTTTCCTTAGACCGGGA
CDH1 P6 F	GCTCTGAGGAGTGGTGCATT
CDH1 P6 R	CAAGACCTAGCCCACCGTTC
CDH1 P7 F	AGACTGAAAGGGAACGGTGG
CDH1 P7 R	CCCACCCCGAAACCTACG
CDH1 P8 F	CTGGGGTCCTCCCCCAAT
CDH1 P8 R	GGTAGATGGAACCGGGTGAC
